# Case Report: Foldable capsular buckle scleral buckling for complex retinal detachment: a case series of three patients

**DOI:** 10.3389/fmed.2026.1945579

**Published:** 2026-10-21

**Authors:** Xiaojing Liu, Wei Wang, Jie Zhang

**Affiliations:** Department of Fundus Diseases, Weifang Eye Hospital, National key Clinical Specialty, Zhengda Guangming Eye Group, Weifang, China

**Keywords:** foldable capsular buckle, pars plana vitrectomy, proliferative vitreoretinopathy, rhegmatogenous retinal detachment, scleral buckling

## Abstract

Complex retinal detachment (RD) is characterized by diverse tear types and extensive detachment, often complicated by proliferative vitreoretinopathy, posing significantly greater therapeutic challenges than simple cases. Conventional scleral buckling often fails to provide adequate support for such tears, and pars plana vitrectomy (PPV), although the current standard for most complex cases, requires intraocular entry with attendant cataract progression in phakic eyes and silicone-oil-related reoperation, as well as prolonged postoperative positioning. This article reports three patients with different types of complex RD (giant retinal tear, horseshoe tear > 60°, and 1PD tear with total detachment), all treated with a foldable capsular buckle (FCB)-based internal-external combined strategy — FCB scleral buckling combined with gas tamponade and laser photocoagulation, with minimally invasive vitrectomy added in Case 3. Postoperative follow-up showed retinal reattachment in all three eyes, visual improvement, and absence of serious complications. These outcomes should be interpreted as the result of an individualized internal–external combined strategy rather than that of FCB alone; the appropriate boundary between this approach and PPV with silicone oil tamponade remains to be defined prospectively. Combined with previously reported cases, the potential role and limitations of FCB within an individualized internal-external combined strategy for complex RD are discussed, to provide a reference for clinical practice.

## Introduction

Retinal detachment (RD) is a serious, vision-threatening condition. It is characterized by the separation of the neurosensory retina from the underlying retinal pigment epithelium. RD results from retinal tears, vitreoretinal traction, and liquefied vitreous passing through the breaks (1). Among the various types of RD, rhegmatogenous retinal detachment (RRD) is the most prevalent. RRD is classified as simple or complex based on the location and extent of detachment, and the presence of complications such as proliferative vitreoretinopathy (PVR) (2). Currently, scleral buckling (SB) remains the preferred treatment for uncomplicated RRD. Pars plana vitrectomy (PPV) has emerged as the standard approach for more complex cases (3). Generally, SB is considered ideal for RRD in young patients with phakic eyes and limited detachment, especially when associated with lattice degeneration or inferiorly located tears (4, 5). However, conventional SB, though an extraocular procedure preserving vitreous anatomy, often fails to provide sufficient and precise support for giant or posteriorly located tears (2). Although PPV can achieve superior outcomes in challenging cases, it has drawbacks. These include accelerated cataract progression in phakic eyes, increased risk of PVR and epiretinal membrane formation, altered oxygen distribution in the vitreous cavity, and high economic costs. In the present report, “complex” RRD refers to detachments featuring one or more of the following: giant retinal tears, very large breaks exceeding the support that conventional rigid explants can provide, total or macula-involving detachment, or grade C PVR — configurations in which conventional SB alone is likely to fail and PPV with silicone oil tamponade is frequently the default choice. In such eyes, whether an foldable capsular buckle (FCB)-based internal-external combined strategy can offer a viable alternative — preserving the crystalline lens in phakic eyes while sparing silicone-oil tamponade and its associated reoperation — remains insufficiently defined.

FCB scleral buckling emerges as a refined modification of conventional SB (6). The FCB is a commercially available product modified from the foldable capsular vitreous body (FCVB) AV-10P model (Vesber, Guangzhou, China). It is made of silicone rubber and consists of a thin vitreous chamber–shaped capsule with a tube-valve system. The device exhibits favorable biocompatibility and weighs approximately 0.3 g. Balanced salt solution (BSS) is injected into the capsule through the tube-valve system, allowing the capsule to expand and take shape. The FCB functions by implantation on the scleral surface, achieving scleral indentation through sterile normal BSS filling, in combination with cryotherapy, gas tamponade, or laser photocoagulation for retinal tear closure (7). Fixation of the FCB requires only one temporary, superficial suture, in contrast to the multiple intrascleral suture passes needed to anchor conventional explants, making the procedure less traumatic and technically simpler than traditional buckling, with less intraoperative and postoperative patient discomfort (1).

While multiple clinical studies have established the feasibility of FCB for uncomplicated RRD, its use in the configurations defined above has not been systematically examined (1, 2). Herein, we report three cases of complex RRD with diverse tear configurations (giant retinal tear, horseshoe tear > 60°, and 1PD tear with total RD) managed with an FCB-based combined strategy. The feasibility of incorporating FCB into an internal-external combined strategy for selected complex detachments is explored, with emphasis on both potential advantages and current limitations.

## Patient selection and informed consent

Preoperative planning included three-dimensional reconstruction of the retinal detachment configuration to determine the optimal FCB implantation position and inflation volume. General selection criteria included: (1) presence of a primary retinal break amenable to focal buckling support; (2) absence of additional breaks in other quadrants requiring extensive circumferential treatment; and (3) PVR of grade C or below, with any grade C proliferation localized to the vicinity of the primary break and judged amenable to counteraction by buckle support combined with gas tamponade and, when necessary, limited vitrectomy.

All three patients were fully informed of the risks and benefits of both PPV with silicone oil tamponade and FCB scleral buckling as alternative surgical options for their complex retinal detachments. Written informed consent was obtained after detailed discussion regarding the anticipated postoperative positioning requirements, potential need for secondary surgeries, and long-term visual outcomes associated with each approach. Specifically, all three patients expressed a strong preference for avoiding silicone oil tamponade and the associated prolonged prone positioning and secondary removal surgery. The decision to proceed with FCB-based external surgery was therefore made through a process of shared decision-making, considering both the anatomical complexity of the detachments and the patients' informed preferences.

## Surgical technique

The surgeries were all performed by the same experienced clinician (Zhang J). Preoperatively, the retrobulbar and supraorbital nerves were anaesthetized with a total of 5 mL of 2% lidocaine and 0.75% bupivacaine (1:1). The conjunctival sac was repeatedly washed with diluted iodophor and saline. Traction sutures were made along the corneal limbus at the meridian line of the retinal break. The bulbar conjunctiva was then incised 5 mm posterior to the limbus, parallel to the limbus and along the direction of the retinal break; the incision measured approximately 4–5 mm. The bulbar conjunctiva and Tenon fascia tissues were separated, and a narrow tunnel was made approximately 12–14 mm long along the episclera posteriorly. A 25-G syringe needle was obliquely inserted into the subretinal space to release the subretinal fluid. The localization of retinal breaks was performed with scleral indentation under an operating microscope. Preplaced sutures of 5–0 nonabsorbable material were placed 4 mm anterior to the posterior margin of the retinal break. The FCB was tested for leakage with sterile air, evacuated, folded, and inserted through the premade tunnel with the spherical surface facing the sclera using intraocular lens platform forceps. Approximately 1 mL of BSS was injected via the tube valve, which was secured with a 5–0 nonabsorbable suture at the tube proximal to the FCB. The position of the retinal break was reconfirmed to be at the apex of the buckle. The conjunctiva with Tenon's capsule incision was closed with sutures, and the tube valve was buried under Tenon's capsule in a transverse direction.

Combined vitreous cavity gas injection, laser photocoagulation, anterior chamber paracentesis and minimally invasive vitrectomy were performed when needed according to the retinal reattachment status.

Tobramycin dexamethasone eye ointment was applied, and the eye was bandaged. Postoperatively, the patient received topical moxifloxacin, prednisolone acetate, tobramycin-dexamethasone ointment, and pranoprofen for anti-inflammatory and anti-infective treatment.

FCB removal is considered once the retina remains attached, all retinal breaks are confirmed closed, and there is no active leakage of subretinal fluid—defined as residual fluid that is fully absorbed, stable, or progressively diminishing on serial follow-up examinations.

Removal of the FCB is a minor procedure performed under local anesthesia in the outpatient clinic. The tube–valve system is exposed through the original conjunctival incision, the intracapsular fluid is released through the tube–valve, and the deflated balloon is then withdrawn.

## Case 1

A 71-year-old male patient was admitted with a chief complaint of “blurred vision in the right eye for over 10 days.” He had a history of hypertension for more than 20 years, diabetes mellitus for more than 20 years, and cerebral infarction for 20 years. Surgical history included cardiac stent placement 7 years ago and cataract surgery in the right eye over 10 years ago. Ophthalmic examination: The uncorrected visual acuity (UCVA) was 0.05 with no improvement on correction in the right eye, and light perception with no improvement on correction in the left eye. Intraocular pressure (IOP) was 4.7 mmHg in the right eye and 12.5 mmHg in the left eye. Slit-lamp examination revealed transparent corneas in both eyes, moderately deep anterior chambers with clear aqueous humor, round pupils with sensitive light reflexes, an intraocular lens *in situ* in the right eye, vitreous opacification, and clear optic disc margins. A 1 disc diameter (PD) sized round retinal tear was observed at the 1:30 peripheral position with grayish elevation of the retina throughout 360 degrees, accompanied by preretinal proliferative membranes consistent with grade C PVR. The left eye showed a dense white mature cataract with an obscured fundus view.

Following admission, a series of ophthalmological auxiliary examinations was conducted (Figure 1a). Admission diagnosis was RRD in the right eye, pseudophakia in the right eye, and complicated cataract in the left eye. On December 29, 2024, the patient underwent SB (FCB), subretinal fluid release and intravitreal gas injection on the right eye. On the first day after the operation, laser photocoagulation of the retina in the right eye was performed (Figure 1b).

**Figure 1:**
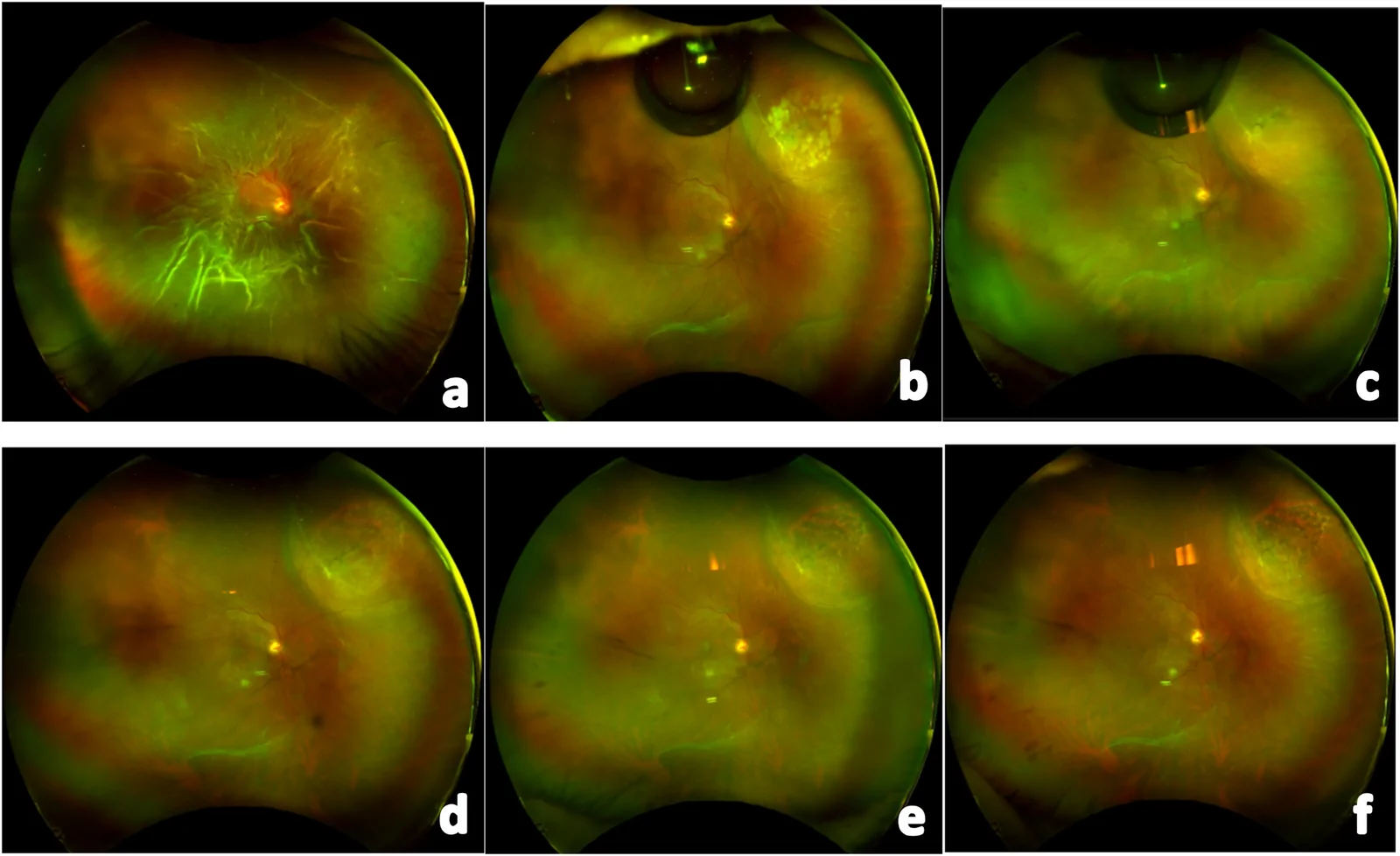
Fundus photography. **(a)**. Total 360° RD involving the macula; **(b)**. Postoperative day 1, the retina was attached, and clear laser photocoagulation spots were visible. Below this, there was vitreous proliferation and peripheral retinal hemorrhage; **c**. Postoperative week 1, minimal residual subretinal fluid; **(d)**. Postoperative month 1, complete absorption of subretinal fluid; (**e,f)**. Postoperative months 3 and 6, stable retinal reattachment.

On the third postoperative day, the UCVA in the right eye measured 0.2, while the best corrected visual acuity (BCVA) was 0.3 with a correction of −1.25 diopter sphere (DS)/−1.00 diopters cylinder (DC) at an axis of 10 degrees. The IOP was 15.3 mmHg. The conjunctival suture in the right eye remained intact. A dome-shaped scleral compression ridge was observed in the upper retina, with the retinal tear situated at the apex of this ridge, surrounded by well-demarcated photocoagulation spots encircling the tear. Intravitreal gas was visible. At the 1-month follow-up, UCVA remained 0.2 and BCVA 0.3, with a refraction of −0.25 DS/−1.00 DC at an axis of 45 degrees. The patient was followed up for one year after the surgery (Figures 1c–f). The UCVA of the right eye was 0.3, which could not be improved by any correction. The IOP was 15.1 mmHg. The retina was well adhered and the laser spots were clear. No FCB extraction procedure was performed.

## Case 2

A 47-year-old male presented with a one-month history of blurred vision in the right eye. He reported a 20-year history of “cerebral insufficiency” but had no other significant ocular or systemic issues. Ophthalmic examination: his UCVA was finger counting at 10 centimeters in the right eye, and his BCVA was 0.15 with a −9.00 DS. The left eye had UCVA of 0.05 and BCVA of 0.6 with −10.25 DS. IOP measured 14.8 mmHg in the right and 16.4 mmHg in the left. Slit-lamp exam showed transparent corneas, moderately deep anterior chambers, clear aqueous humor, round pupils with sensitive light reflexes, dense lenses, and mild vitreous opacity in both eyes. Fundus exam in the right eye found a well-defined, normally colored optic disc, a peripapillary choroidal atrophy arc, and a 20-PD horseshoe retinal tear in the temporal quadrant with grayish elevation of the temporal retina involving the macula. The left eye showed a well-defined optic disc, a peripapillary choroidal atrophy arc, a tigroid fundus, and no foveal reflex.

Following admission, comprehensive ophthalmological auxiliary examinations were conducted, including retinal optical coherence tomography (OCT) (Figure 2a), ocular B-scan ultrasonography (Figure 2b), and fundus photography (Figure 2c). The patient was diagnosed with RRD in the right eye and refractive error in both eyes. On June 18, 2025, the patient underwent FCB scleral buckling, subretinal fluid release and intravitreal gas injection on the right eye. Retinal laser photocoagulation was performed on postoperative days 1 and 5 to effectively seal the retinal tear.

**Figure 2:**
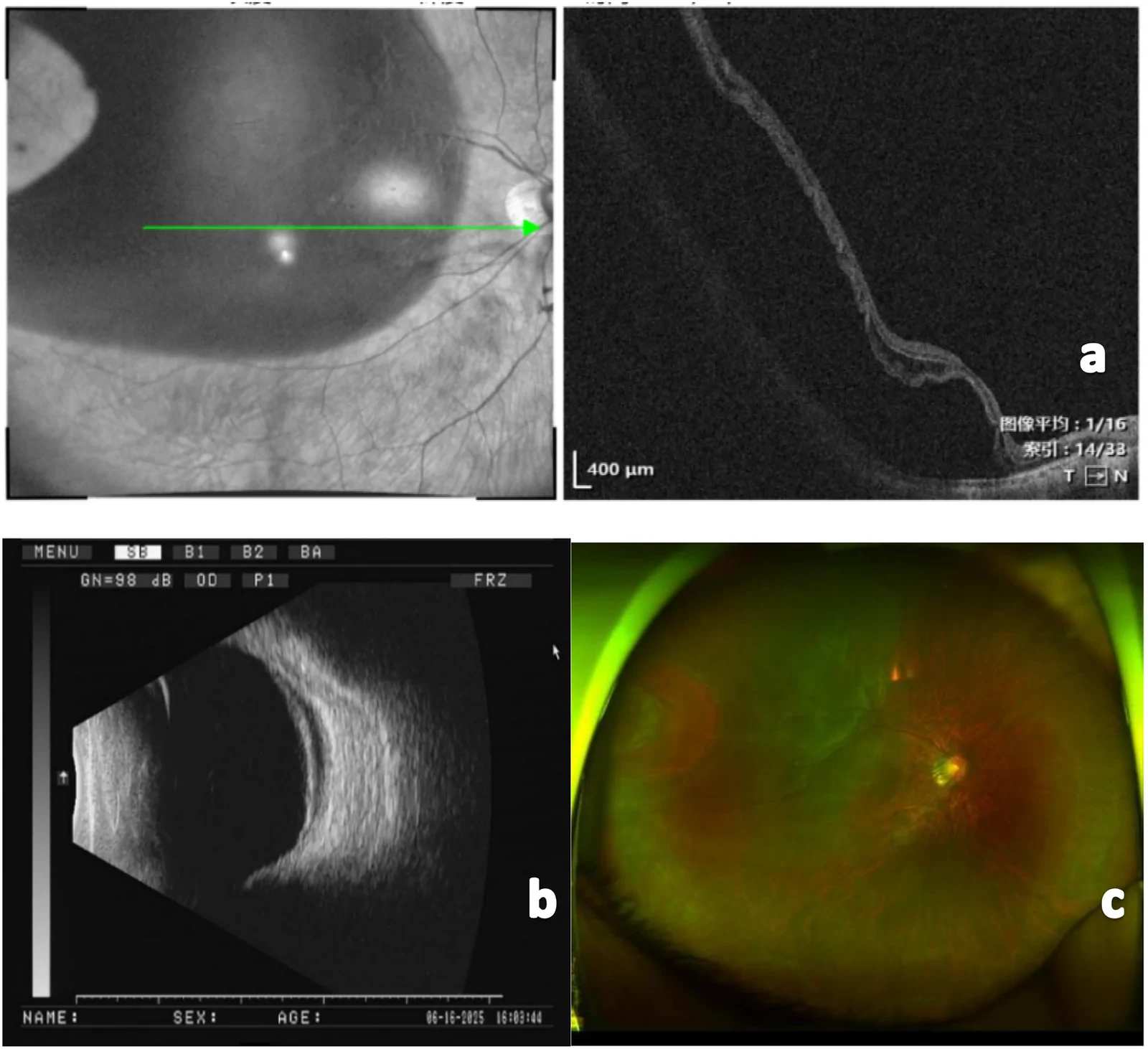
**a**. Retinal OCT revealed RD extending to the macular region, with subretinal fluid accumulation. **b**. B-ultrasound scan showing RD pre-surgery. **c**. Fundus photography: In the right eye found a well-defined, normally colored optic disc, a peripapillary choroidal atrophy arc, and a 20-PD horseshoe retinal tear in the temporal quadrant with grayish elevation of the temporal retina involving the macula.

On the fifth postoperative day, the UCVA in the right eye was measured at 0.04, while the BCVA was 0.25, with a correction of −9.25 DS/−3.25 DC at an axis of 75 degrees. The IOP was 18.3 mmHg. The conjunctival suture in the right eye remained intact. A dome-shaped scleral compression ridge was observed in the upper retina, with the retinal tear situated at the apex of this ridge and surrounded by well-demarcated photocoagulation spots. A small amount of gas persisted in the vitreous cavity (Figure 3). During the postoperative follow-up, comprehensive ophthalmic evaluations were conducted, including ocular B-ultrasound (Figure 4a), fundus photography (Figure 4b), and retinal OCT (Figure 4c). Immediately before FCB removal (postoperative day 50), UCVA was 0.04 and BCVA was 0.6 with a correction of −9.00 DS/−3.00 DC at an axis of 75 degrees. The FCB was then removed as described above. One month after FCB removal, the UCVA in the right eye was 0.04, the BCVA was 0.6 with a correction of −9.00 DS/−3.00 DC at an axis of 75 degrees. The IOP was 17.6 mmHg. The retina remained flat with complete absorption of the subretinal fluid. At the last follow-up, 6 months after FCB removal, UCVA was 0.05 and BCVA remained 0.6; the retina remained attached with no recurrent detachment.

**Figure 3:**
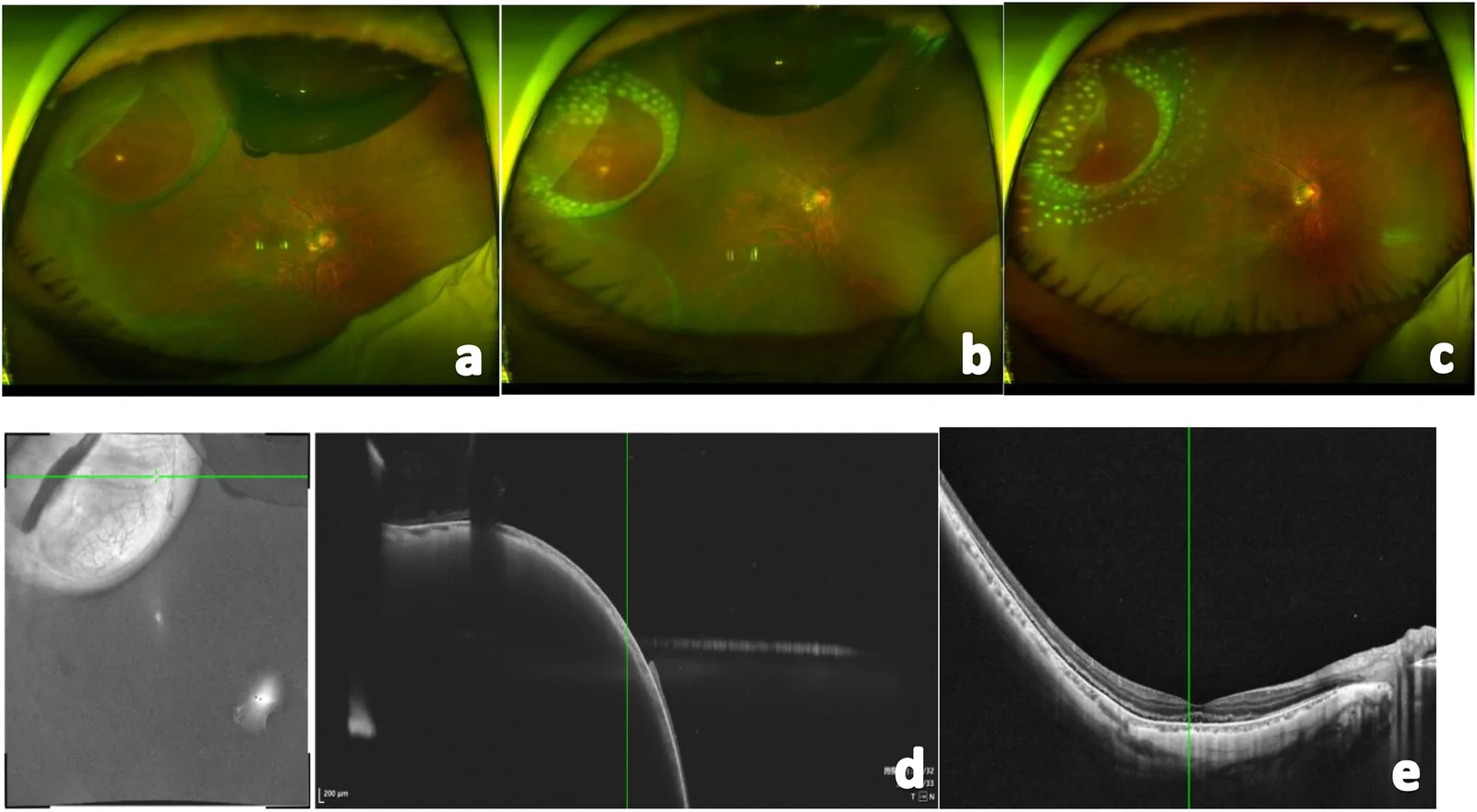
Fundus photography: **(a)**. Postoperative day 1, retinal reattachment; **(b,c)**. Clear laser photocoagulation spots postoperatively. Retinal OCT: **(d)**. Retinal tear located at the highest position with reattached retina; (**e)**. Subretinal fluid in the macular region.

**Figure 4:**
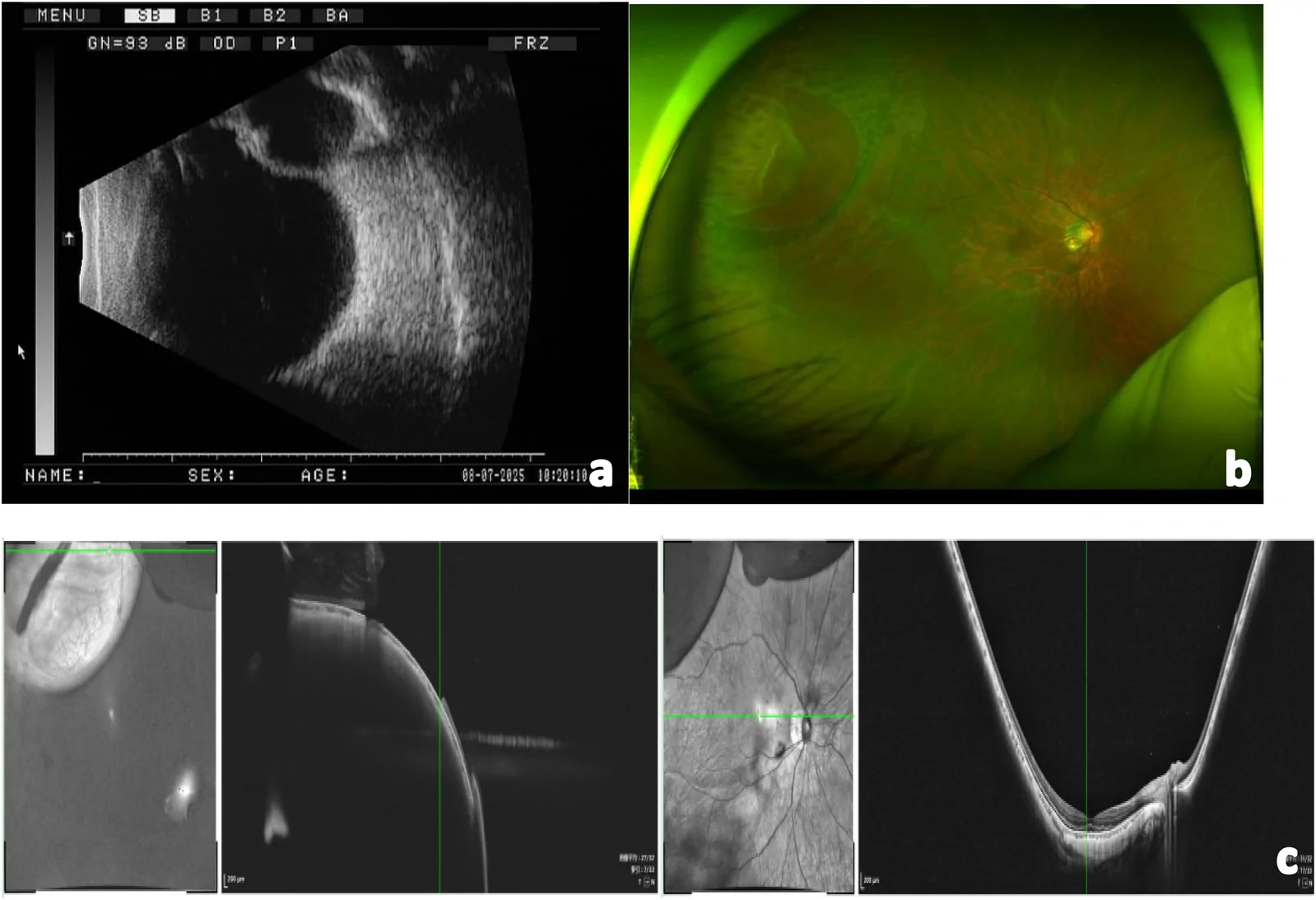
**(a)**. Post-operative B-ultrasound scan showing FCB's evident pressure on the sclera. **(b)**. Fundus image showed clear, distinct photocoagulation spots with well-reattached retina, and the scleral compression ridge was visible above the retina with the tear located at its highest point. **(c)**. Postoperative OCT demonstrated the tear at the uppermost position with well-apposed surrounding retina, minimal residual subretinal fluid, and complete macular reattachment.

## Case 3

A 41-year-old male patient presented with a one-month history of a visual field defect in the left eye. The patient had no significant medical history. Ophthalmic examination: The UCVA for the right eye was 0.1, with a BCVA of 1.0 using a correction of −3.75 DS/−0.50 DC at an axis of 5 degrees. For the left eye, the UCVA was 0.12, and the BCVA was 1.0 with a correction of −2.50 DS/−0.75 DC at an axis of 5 degrees. IOP was measured at 12.6 mmHg in the right eye and 11.6 mmHg in the left eye. Slit-lamp examination indicated transparent corneas, moderately deep anterior chambers, clear aqueous humor, round pupils with sensitive light reflexes, dense lenses, and mild vitreous opacity in both eyes. Fundoscopic examination of the right eye revealed a well-defined optic disc with normal coloration, a peripapillary choroidal atrophy arc, a band-shaped tear approximately 8 PD at the 12 o'clock position, and a 1 PD round tear at the 1 o'clock position. The left eye exhibited a well-defined optic disc with normal coloration, a peripapillary choroidal atrophy arc, a giant tear approximately 30-PD at the 12 o'clock position accompanied by surrounding retinal folds and vitreous proliferation, a grayish elevation of the retina from 10:00 to 1:00, and a 1/2 PD round tear at the 4:30 position.

Following admission, a series of ophthalmological auxiliary examinations was conducted, including fundus photography (Figure 5a), ocular B-scan ultrasonography (Figure 5b), and retinal OCT (Figure 5c). The patient was diagnosed with RRD in the left eye and retinal tears in the right eye. On August 5, 2025, the patient underwent binocular retinal laser photocoagulation. The following day, SB with FCB implantation, anterior chamber paracentesis, subretinal fluid release, minimally invasive vitrectomy, and intravitreal gas injection were performed on the left eye. On August 7, 2025, additional retinal laser photocoagulation was administered to the left eye.

**Figure 5:**
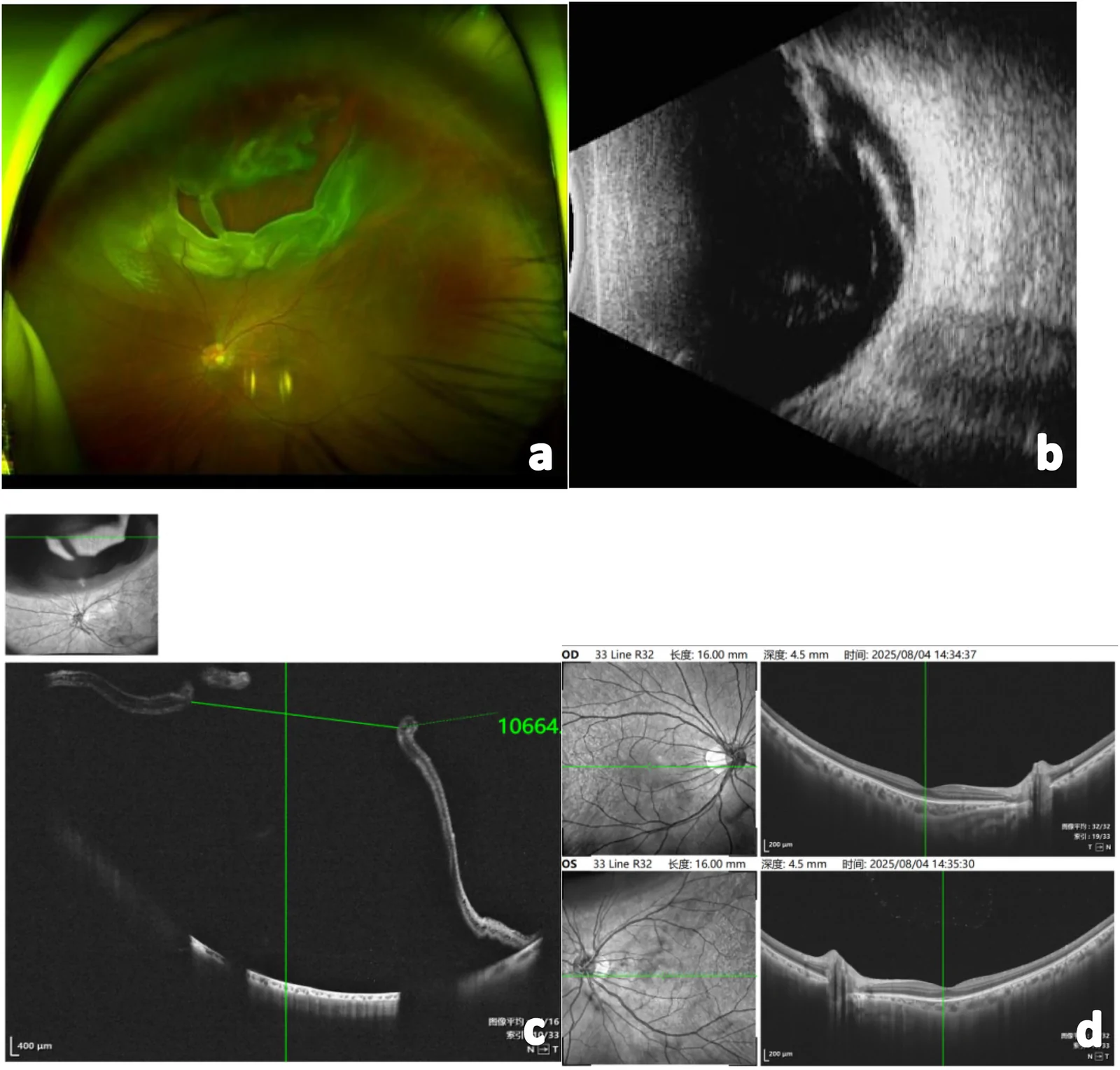
**(a)**. Fundus photograph: A giant retinal tear approximately 30-PD in size, located at the 12 o'clock position, accompanied by surrounding retinal folds and vitreous proliferation. (**b)**. B-ultrasound scan showing RD pre-surgery. **(c)**. Retinal OCT shows an unaffected macula. There is a partial absence of the neuroepithelial layer, measuring approximately 10,664 micrometers in diameter. **(d)**. The macular area of the retina is well attached.

On the second postoperative day, the UCVA in the left eye measured 0.15, while the BCVA was 0.8 with a correction of −4.50 DS. The IOP was recorded at 13.6 mmHg. The conjunctival suture in the left eye remained intact, and a small amount of gas persisted in the vitreous cavity. A dome-shaped scleral compression ridge was observed in the upper retina, with the retinal tear situated at the apex of this ridge and surrounded by well-demarcated photocoagulation spots (Figure 6). Comprehensive ophthalmic evaluations were conducted during the postoperative follow-up period, including fundus photography (Figure 7a), OCT of the retina (Figure 7b), and ocular B-ultrasound (Figure 7c). Immediately before FCB removal (postoperative day 40), UCVA was 0.2 and BCVA was 1.0 with a correction of −4.00 DS. The FCB was then removed as described above. At the last follow-up, 5 months after FCB removal, UCVA of 0.15 and the BCVA had improved to 1.0 with a correction of −4.00 DS in the left eye, the retinal remained attached with complete absorption of subretinal fluid and no recurrent detachment (Figure 8).

**Figure 6:**
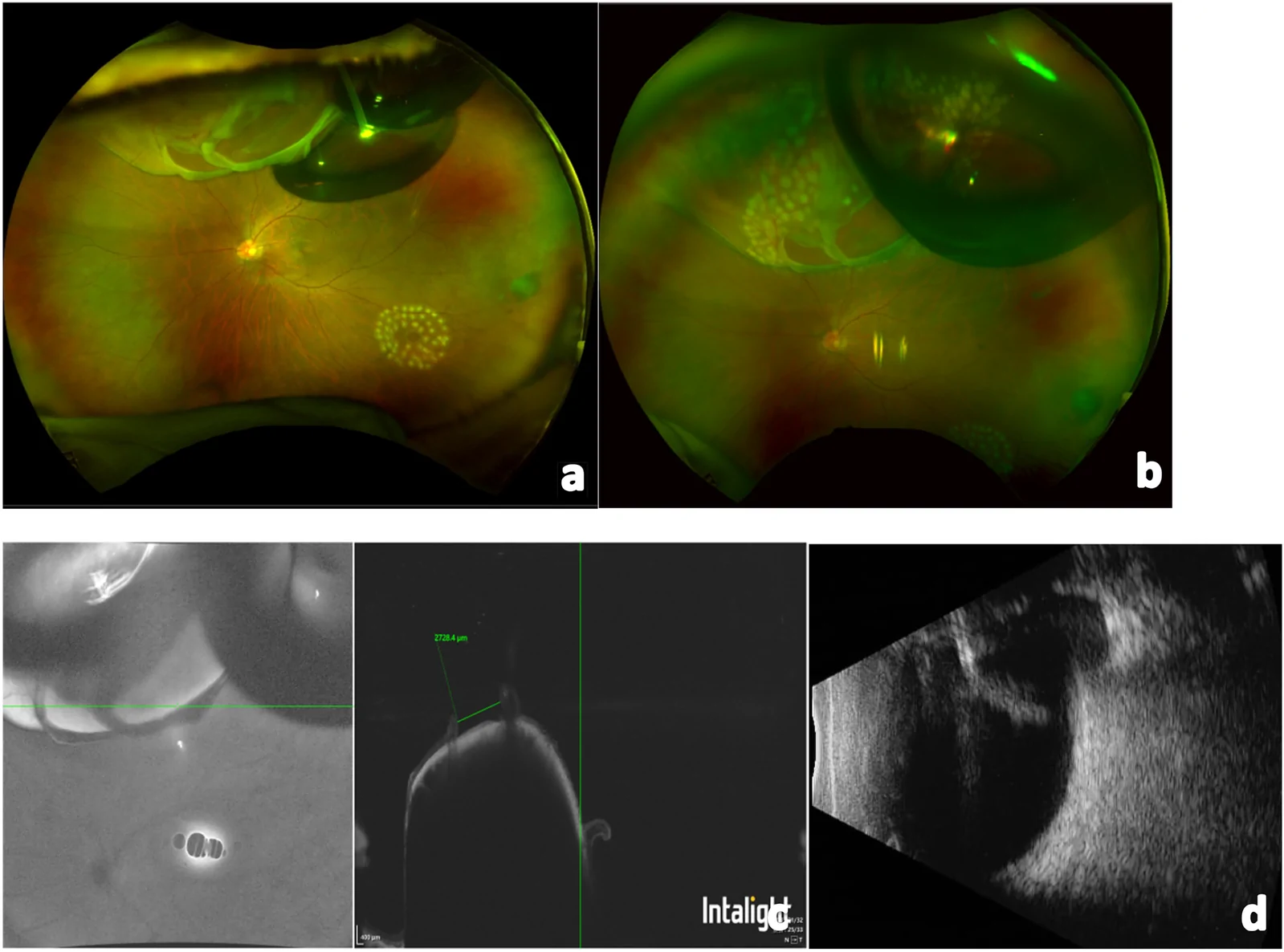
Fundus photography: **(a)**. Postoperative day 1, retinal reattachment; **(b)**. Clear laser photocoagulation spots postoperatively. **(c)**. Retinal OCT shows a partial absence of the neuroepithelial layer, measuring approximately 2,728.4 micrometers in diameter. Retinal tear located at the uppermost position with reattached retina. **(d)**. Post-operative B-ultrasound scan showing FCB's evident pressure on the sclera.

**Figure 7:**
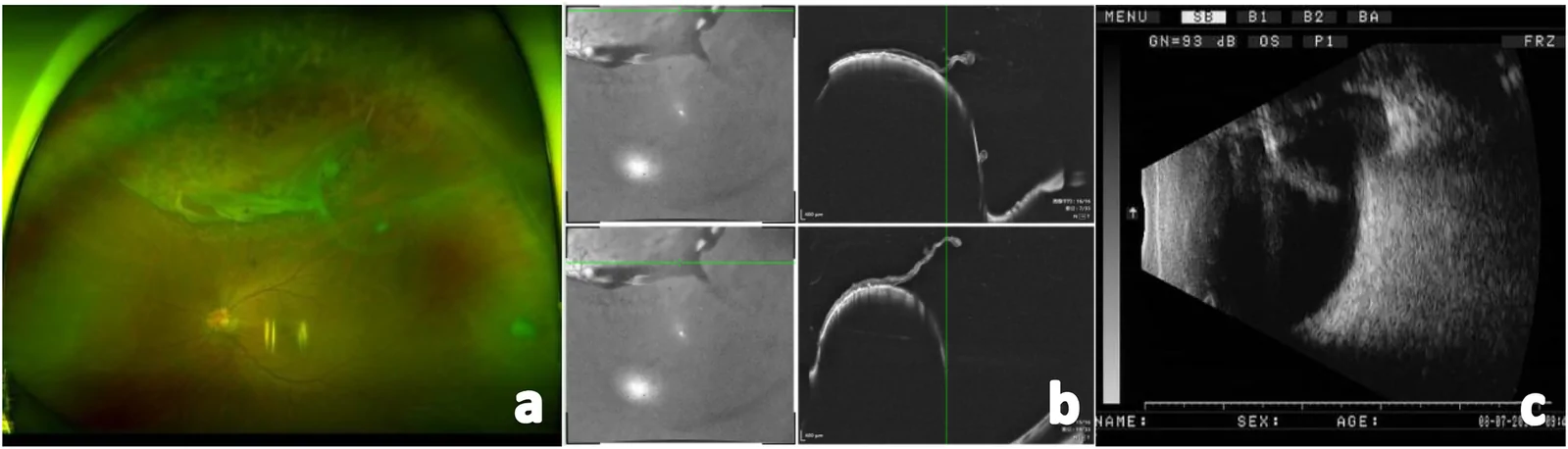
**(a)**. Fundus image showed clear, distinct photocoagulation spots with well-reattached retina, and the scleral compression ridge was visible above the retina with the tear located at its highest point. **(b)**. Retinal OCT: The retinal tear above the left eye was located on the compression ridge, and the retinal neuroepithelial layer around the tear was well attached to the pigment epithelial layer. **(c)**. Post-operative B-ultrasound scan showing FCB's evident pressure on the sclera.

**Figure 8:**
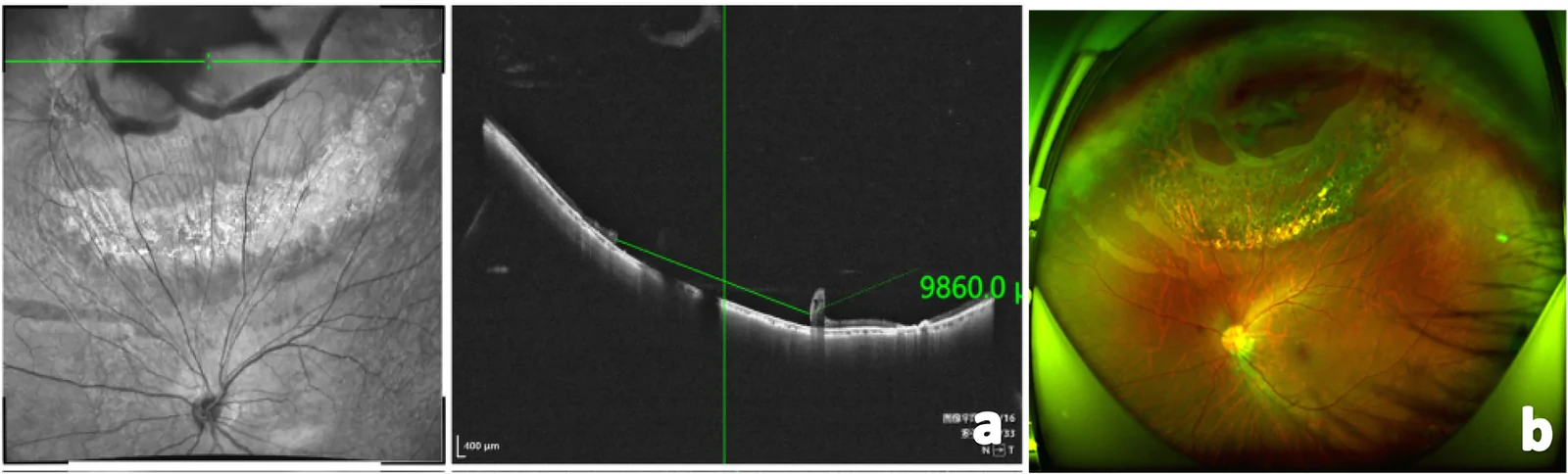
**(a)**. Retinal OCT: The subretinal fluid above the left eye was completely absorbed and fitted well. The diameter of the tear was about 9,860 um. **(b)**. Fundus image at 5 months after FCB removal, showing the well-reattached retina.

The clinical timelines of the three patients are summarized in Table 1.

**Table 1:** Clinical timeline of the three patients.

Timeline	Case 1	Case 2	Case 3
Presenting complaint	Blurred vision in the right eye for over 10 days	Blurred vision in the right eye for 1 month	Visual field defect in the left eye for 1 month
Preoperative findings	Total 360° RD; 1-PD round tear at 1:30; grade C PVR	horseshoe tear > 60° in the temporal quadrant involving the macula	30-PD giant tear at 12:00 with an inverted flap; 1/2-PD round break at 4:30; RD from 10:00 to 1:00; grade C PVR
Surgery	2024–12–29: FCB implantation + subretinal fluid release + intravitreal gas tamponade	2025-06-18: FCB implantation + subretinal fluid release + intravitreal gas tamponade	2025-08-06: FCB implantation + anterior chamber paracentesis + subretinal fluid release + minimally invasive vitrectomy + intravitreal gas tamponade
Postoperative laser	Postoperative day 1	Postoperative days 1 and 5	Postoperative day 1
FCB removal	Retained *in situ*	Postoperative day 50	Postoperative day 40
Last follow-up	12 months postoperatively	7 months postoperatively (6 months after FCB removal)	6 months postoperatively (5 months after FCB removal)
Key follow-up visits	Postoperative day 1, 1 week, 1 month, 3, 6, and 12 months	Postoperative day 1, pre-removal, 1 week post-removal, 6 months postoperatively	Postoperative day 1, pre-removal; 1 week post-removal; 5 months postoperatively

## Discussion

Previous studies have reported the safety and efficacy of FCB scleral buckling in the management of simple RRD, small retinal breaks (<6 mm), and selected single giant tears. This case series incorporated the use of FCB in challenging RDs with large and complex tears, with the aim of exploring its potential role within an individualized internal-external combined surgical strategy.

All three patients achieved favorable anatomical and functional outcomes with durable retinal stability. Complete retinal reattachment was accomplished in all eyes without recurrent detachment. Case 1 completed 12 months of follow-up with the FCB retained *in situ*, while Cases 2 and 3 underwent elective FCB removal at 50 and 40 days postoperatively, respectively, followed by regular surveillance for an additional 5–6 months. Case 1 is of particular clinical importance, as it presented with total 360° RD, a condition historically regarded as a relative contraindication for isolated SB. It should be acknowledged that this eye had grade C PVR, extending beyond the grade-based indications used in previous FCB studies; the proliferation was, however, localized to the vicinity of the primary break and could be counteracted by the combined strategy. The stable 12-month reattachment achieved here is consistent with the feasibility of including such localized grade C proliferation within the selection criteria, whereas eyes with extensive or circumferential proliferation, in which focal buckling support cannot reliably counteract the traction, are not considered candidates for this strategy and are instead managed with PPV and silicone oil tamponade. Given the patient's advanced age, substantial vitreous liquefaction would be anticipated; together with the total detachment, a 1PD tear, and low baseline IOP (4.7 mmHg), this placed the eye outside the established indications for pneumatic retinopexy and would compromise the efficacy of gas tamponade alone. In this context, FCB scleral buckling provides essential external mechanical support—its broad spherical indentation generates a smooth pressure ridge that directly supports the detached retina and facilitates subretinal fluid drainage—thereby complementing rather than replacing the role of gas tamponade (8). Postoperative observation of a dome-shaped scleral compression ridge with the tear situated at its apex confirmed the sustained physical contribution of the buckle. Successful long-term reattachment in this complex case highlights the value of combining FCB with adjunctive gas tamponade for refractory RRD. The decision to retain the FCB was a deliberate one: given the preretinal proliferation, buckle removal would risk leaving residual traction unopposed, whereas the retained indentation provides continued mechanical support. Moreover, the FCB was well tolerated throughout the 12-month follow-up, without diplopia or other buckle-related complications; the FCB was therefore left *in situ*, in view of the patient's age and overall ocular condition.

Case 2 presented with a horseshoe tear > 60° involving the macula in a highly myopic eye (−9.00 DS), and a one-month disease course. High myopia has traditionally been regarded as a relative contraindication to SB due to long axial length, thin sclera, and posteriorly located tears (9). The thin sclera in such eyes increases the risk of perforation during deep scleral suturing for conventional buckles; FCB fixation, however, requires only an episcleral tunnel and a single superficial suture, minimizing this risk. Moreover, by restoring posterior pole anatomy through external compression without intraocular entry, FCB avoids the direct mechanical insults to the macula associated with vitrectomy, while also preserving the natural lens and preoperative refractive status—thereby averting the near-certain cataract progression and additional intraocular procedure that would follow PPV with silicone oil tamponade (10). Despite these unfavorable anatomical features, the retina was successfully reattached and the patient attained promising visual recovery.

Case 3 presented a giant retinal tear of approximately 30-PD at the 12 o'clock position with posteriorly inverted flaps and grade C PVR. This configuration is particularly challenging because the inverted posterior flap generates persistent anterior-posterior traction vectors that resist spontaneous reattachment and predispose to redetachment even after successful initial repair (2). Minimally invasive vitrectomy was therefore performed to release PVR-related vitreous traction on the posterior lip of the tear, relieve the inverted flap, and facilitate intraoperative manipulation. However, vitrectomy alone does not provide sustained scleral support at the tear margin; in giant retinal tears, the elastic recoil of the posterior flap creates ongoing traction during the postoperative adhesion-forming period that gas tamponade alone cannot reliably counteract. The FCB was therefore implanted to provide a broad, spherical scleral compression ridge that mechanically opposes this recoil, maintains the tear edge in apposition to the retinal pigment epithelium, and reduces tangential traction at the tear margins during the critical period of retinal adhesion formation (11). Early postoperative OCT showed persistent subretinal fluid at the tear margin with incomplete apposition of the neurosensory retina to the RPE, which was not indicative of surgical failure but rather reflected chronic tissue stiffness and contracture of the long-standing inverted flap, as well as slow absorption of viscous subretinal fluid. In a prospective series, OCT-detectable residual subretinal fluid persisted in over 80% of eyes at one month after anatomically successful buckling and required months to resolve completely (12, 13). By postoperative day 40, complete retinal adhesion was achieved, and subsequent FCB removal confirmed durable retinal stability without recurrent detachment.

It is important to emphasize that the FCB was not evaluated as a standalone intervention in any of the three cases. All patients received concomitant gas tamponade and laser photocoagulation, and Case 3 underwent additional vitrectomy. Therefore, the observed anatomical outcomes cannot be attributed to the FCB buckle in isolation, and this series should be interpreted as a report of internal-external combined surgical management rather than an assessment of FCB efficacy *per se*.

Collectively, postoperative findings from B-scan ultrasonography, fundus photography and sequential BCVA measurements confirmed that FCB provided sustained, stable scleral compression with reliable positional stability, without displacement induced by routine eye movement (8). Building on prior evidence supporting FCB safety and efficacy in simple, uncomplicated RRD, the present series explores the potential applicability of this modified technique to highly complex, challenging RD. Concurrently, FCB scleral buckling has recently been successfully applied in pregnant patients with RRD, in whom avoidance of general anesthesia, prolonged prone positioning, and oculocardiac reflex stimulation is particularly desirable (14, 15) — suggesting that the potential applications of this technique may warrant further investigation across increasingly challenging clinical scenarios.

Compared with traditional SB, which requires repeated extraocular muscle traction and carries the risk of pain and oculocardiac reflex, FCB scleral buckling minimizes extraocular manipulation, reduces surgical trauma, and is associated with less postoperative discomfort. Its 13-mm spherical design generates a broad, smooth pressure ridge with uniform force distribution across the indented sclera (1, 2), offering three advantages over conventional segmental buckles: (1) a substantially larger indentation surface that supports extensive tears, multiple adjacent breaks, and posterior breaks; (2) a smoother pressure profile that reduces fish-mouthing, localized choroidal ischemia, and retinal surface tension to promote reattachment (2); and (3) a foldable, injectable design allowing implantation through a small conjunctival incision, placement beneath the extraocular muscles, and elective extraction once retinal adhesion has matured—thereby avoiding the long-term complications of permanent buckles such as extrusion, infection, and induced myopia (16). The valve system additionally permits intraoperative titration of indentation force. In this series, by combining FCB scleral buckling with intravitreal gas tamponade, retinal laser photocoagulation, and subretinal fluid drainage, with Case 3 additionally undergoing minimally invasive vitrectomy to address vitreous traction, we established a comprehensive internal-external combined strategy. Retinal laser photocoagulation was selected over cryotherapy to minimize the inflammatory response and blood-retinal barrier breakdown associated with cryotherapy, which may increase PVR risk—particularly undesirable in complex cases with preexisting proliferative changes (17). Postoperative laser also allowed precise targeting after FCB stabilization and retinal reattachment, avoiding the imprecision of intraoperative cryotherapy given the altered retinal topography during buckle adjustment. We acknowledge that this staged approach necessitates an additional procedure, but the gains in precision and reduced PVR risk justify this trade-off.

In the present series, the primary retinal breaks were located superonasally (1:30) in Case 1, superiorly (12 o'clock) in Case 3, and temporally in Case 2. For superior breaks, intraocular gas tamponade allows the break to be positioned at the highest point of the globe postoperatively, thereby maximizing the internal tamponade effect and facilitating chorioretinal adhesion formation; temporal breaks can likewise be effectively tamponaded with appropriate lateral posturing. However, this anatomical distribution should not be interpreted as a limitation of FCB to superior pathology. The broad, spherical indentation surface of the FCB generates a uniform pressure ridge that is theoretically capable of supporting retinal breaks in any quadrant, provided that accurate localization and adequate buckle height are achieved (16). Indeed, previous reports have described successful FCB placement for tears located in the temporal, nasal, and inferior quadrants, with no inherent mechanical restriction based on tear location (2, 6). In our clinical practice, FCB has been applied to tears in various positions; the present series simply reflects the coincidental superior and temporal location of these particular complex cases. The efficacy of FCB combined with gas tamponade for inferior tears may be influenced by gravitational effects on gas bubble dynamics, and this specific scenario warrants dedicated investigation in future studies.

In this study, we found that the best corrected visual acuity after FCB scleral buckling surgery was improved compared with that before surgery, but the patient's refractive error also changed to a certain extent. Case 1, with the FCB retained *in situ*, showed persistent myopic shift and residual astigmatism throughout follow-up. Case 2 exhibited induced astigmatism, and Case 3 exhibited an increased myopic spherical equivalent; although these changes partially improved over the course of follow-up, the refraction did not fully return to preoperative levels, even after FCB removal (6). Serial refraction data obtained preoperatively, with the buckle *in situ*, and at the last follow-up visit are summarized in Table 2. In traditional SB surgery, the sutured silicone buckle will exert pressure on the eyeball in different directions, especially when the retinal tear is located below the rectus muscle, which may potentially cause eyeball deformation and lead to changes in refractive status (18, 19). In FCB scleral buckling surgery, the liquid in the balloon used can be removed after the retinal tear is closed, which helps to minimize the effect on the degree of astigmatism in the affected eye. This suggests that even after the balloon fluid is removed, scleral compression during FCB implantation may still transiently or persistently alter the refractive state. Long-term refractive outcomes following FCB scleral buckling require further study.

**Table 2:** Refractive outcomes of the three patients.

	Preoperative			Buckle *in situ*			Last follow-up		
	UCVA	BCVA	Refraction	UCVA	BCVA	Refraction	UCVA	BCVA	Refraction
Case 1	0.05	0.05	0.00	0.2	0.3	−0.25/−1.00 × 45°	0.3	0.3	−0.50/−1.00 × 45°
Case 2	FC/10cm	0.15	−9.00	0.04	0.6	−9.00/−3.00 × 75°	0.05	0.6	−9.00/−3.00 × 75°
Case 3	0.12	1.0	−2.50/−0.75 × 5°	0.2	1.0	−4.00	0.15	1.0	−4.00

FC, finger counting; Refraction values are given as diopter sphere (DS)/diopter cylinder (DC) × axis in degrees; entries without a cylinder value indicate a spherical correction only. The “Buckle *in situ*” column shows values obtained 1 month after FCB implantation in Case 1, on postoperative day 50 in Case 2 (immediately before FCB removal), and on postoperative day 40 in Case 3 (immediately before FCB removal). The “Last follow-up” column shows values obtained 12 months postoperatively with the FCB retained *in situ* in Case 1, 6 months after FCB removal in Case 2, and 5 months after FCB removal in Case 3.

No serious intraoperative or postoperative complications were observed in this series.

The only procedure-related adverse effect was an anticipated refractive shift, which partially resolved over the course of follow-up in Cases 2 and 3 and persisted with the FCB retained in Case 1. However, potential risks documented in previous studies include transient intraocular pressure elevation, balloon displacement or leakage, conjunctival erosion over the tube valve, transient diplopia, and scleral perforation during suture placement in eyes with thinned sclera (1). The absence of complications in this small series likely reflects careful patient selection; larger cohorts are needed to fully establish the safety profile of FCB in complex retinal detachments.

Overall, these preliminary findings suggest that an FCB-based internal-external combined strategy may be feasible for appropriately selected cases of complex RD. The FCB's contribution lies in providing sustained external mechanical support without requiring intraocular silicone oil tamponade or prolonged prone positioning. While PPV with silicone oil can achieve high anatomical success rates in similar complex detachments, it obligates a second operation for oil removal with its attendant risks. In patients who wish to avoid oil-related re-operation or cannot comply with prolonged postoperative prone positioning, FCB-based external buckling combined with gas tamponade may offer a viable alternative. Whether this approach translates into expanded surgical indications beyond conventional SB boundaries remains to be established prospectively.

## Limitations

This study has several limitations inherent to its case-series design. The small sample size and absence of a control group preclude any assessment of effectiveness or superiority relative to conventional SB or PPV. The follow-up duration was relatively short, and all primary tears in this series were located in the superior or temporal quadrants; the efficacy of this approach for breaks in other quadrants and for complex tear configurations beyond those presented here remains to be investigated. In addition, the inclusion of localized grade C PVR within the selection criteria reflects the experience of this small series and requires prospective validation. The decision to offer the combined FCB strategy rather than primary PPV with silicone oil was individualized and partly preference-based, introducing potential selection bias; the criteria delineating which complex detachments are suitable for this approach remain to be refined.

## Conclusion

In conclusion, this case series provides preliminary evidence that an FCB-based internal-external combined strategy may be feasible for appropriately selected cases of complex RD that are traditionally managed with PPV. The three patients, presenting with a total detachment with a 1PD tear, a horseshoe tear exceeding 60°, and a giant retinal tear of approximately 30-PD, respectively, all achieved anatomical reattachment following combined treatment with FCB, gas tamponade, laser photocoagulation, and adjunctive procedures as indicated. For selected patients, this approach may offer an opportunity to preserve the vitreous body and avoid silicone oil tamponade with its associated re-operation. However, given the limitations inherent to this small, uncontrolled case series, the observed outcomes cannot be attributed solely to the FCB buckle, and no assessment of effectiveness, safety, or superiority over conventional SB or PPV is possible. Further prospective, controlled studies with larger cohorts are warranted to better define the role of, and patient selection criteria for, FCB in the management of complex RD.

## Data Availability

The original contributions presented in the study are included in the article/Supplementary Material, further inquiries can be directed to the corresponding author.
